# Phs1 and the Synthesis of Very Long Chain Fatty Acids Are Required for Ballistospore Formation

**DOI:** 10.1371/journal.pone.0105147

**Published:** 2014-08-22

**Authors:** Giuseppe Ianiri, Ritika Abhyankar, Akio Kihara, Alexander Idnurm

**Affiliations:** 1 School of Biological Sciences, University of Missouri-Kansas City, Kansas City, Missouri, United States of America; 2 Dipartimento di Agricoltura, Ambiente e Alimenti, Facoltà di Agraria, Università degli Studi del Molise, Campobasso, Italy; 3 Pembroke Hill School, Kansas City, Missouri, United States of America; 4 Laboratory of Biochemistry, Faculty of Pharmaceutical Sciences, Hokkaido University, Sapporo, Hokkaido, Japan; 5 School of Botany, University of Melbourne, Victoria, Australia; University of Wisconsin – Madison, United States of America

## Abstract

The production and dissemination of spores by members of the fungal kingdom is a major reason for the success of this eukaryotic lineage in colonizing most terrestrial ecosystems. Ballistospores are a type of spore produced by basidiomycete fungi, such as the mushrooms and plant pathogenic rusts. These spores are forcefully discharged through a unique liquid-drop fusion mechanism, enabling the aerosolization of these particles that can contribute to plant disease and human allergies. The genes responsible for this process are unknown due to technical challenges in studying many of the fungi that produce ballistospores. Here, we applied newly-developed techniques in a forward genetic screen to identify genes required for ballistospore formation or function in a tractable red yeast, a species of *Sporobolomyces*. One strain bearing a mutation in the *PHS1* gene was identified as a *mirror* mutant. *PHS1* encodes 3-hydroxyacyl-CoA dehydratase required for the third step in very long chain fatty acid biosynthesis. The *Sporobolomyces PHS1* gene complements the essential functions of a *S. cerevisiae phs1* mutant. The *Sporobolomyces phs1* mutant strain has less dehydratase activity and a reduction in very long chain fatty acids compared to wild type. The mutant strain also exhibits sensitivity to cell wall stress agents and loss of shooting due to a delay in ballistospore formation, indicating that the role of Phs1 in spore dissemination may be primarily in cellular integrity.

## Introduction

Life on land without the convenience of an aqueous environment presents many challenges to organisms, one of which is how to spread from one location to another to find new nutrient sources or mating partners. The three macroscopic kingdoms of eukaryotes have solved this problem in different ways. For instance, animals are able to move physically. Plants disperse by pollen and seed, leading to the elaborate methods present today for pollination or encasing seed within fruits to attract animals. The third group, the fungi, often use spores for dispersal in the air, requiring physical force to launch the spores away from the colony or fruiting body.

**Figure 1:**
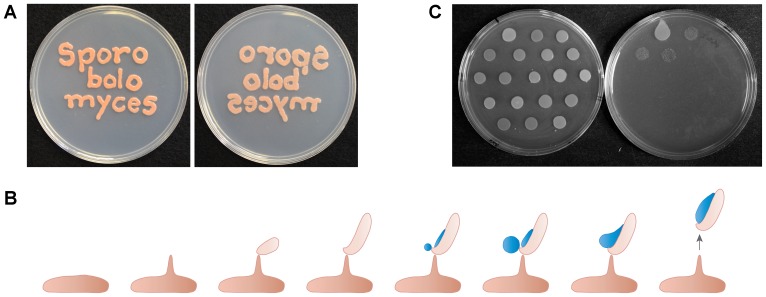
The “mirror” capability of ballistospore-forming yeast species. (A) The plate on the left was inoculated with *Sporobolomyces* sp. wild type strain IAM 13481, and inverted onto an uninoculated plate on the right. (B) Diagram of the steps in the formation of a ballistospore from a yeast cell, based on [2], [10]. (C) The 18 *mirror* mutants isolated in this study growing on the top plate (left) and their replication to a bottom plate (right). The wild type strain is in the top left hand corner of the bottom plate.

Fungi have evolved mechanism to disperse spores on independent occasions. These mechanisms include firing the ascospores from fruiting structures in Ascomycetes, the water canon of *Pilobolus* spp., water splash dispersal in the Nidulariaceae (bird's nest fungi) and a springboard in *Sphaerobolus* spp. [1]. One spore type that is unique to the basidiomycetes is the ballistospore (Fig. 1). Ballistospores can be produced either asexually (ballistoconidia) or sexually (basidiospores). The process has attracted interest for more than a century, with ballistospore-forming yeasts termed “mirror” yeasts, taking this name by being able to produce a mirror image of the culture from one Petri dish on an uninoculated second dish (Fig. 1A). For instance, noted mycologist A.H.R. Buller (1874–1944) dedicated a considerable part of his career to research on ballistospore formation in the red yeasts [2], and the characteristic liquid that forms on the ballistospore is termed “Buller's drop” as a consequence. More recent analyses, including high-speed video photography, reveals that spore release involves a fusion event between Buller's and a second drop of liquid that forms on the side of the ballistospore [3], [4] (Fig. 1B).

Ballistospores are prevalent particles in the air during certain times of the year when their associated species are sporulating, and when these spores form the majority of bioaerosols [5], [6]. Two important influences that ballistospores have on humans are their roles in the life cycles of the rust fungi and as airborne particles that cause allergies in people. First, the rusts are obligate plant parasites, i.e. they cannot be cultured in vitro. They cause disease in thousands of plant species, the most economically serious being rusts of grain crops. Second, basidiomycete spores are a common cause of allergic asthma. Rates of affected individuals vary depending on country, environment and season. The limited access to antigens hinder detection of reactivity to allergens in patients, leading to underestimates in incidence. Nevertheless, metadata analyses of clinical studies indicate that 3 to 35% of asthma patients react to basidiomycetes [7]–[9]. The sources of these allergens are ballistospores fired from fungi, and that circulated in the air until inhaled by susceptible people.

The process of ballistospore biosynthesis requires intricate cell morphogenesis and developmental timing, with highly polarized growth, partitioning of the spore cell into different areas, and asymmetrical release of sugars to trigger the formation of Buller's and the adaxial drops of liquid (Fig. 1B). Another striking feature of ballistospores is the force generated for propulsion. This is of relevance because spores fired from mushrooms must avoid impact on the adjacent side of the gill. In contrast, spore release from red yeasts is optimized for maximum dispersion in air currents [10]. The main forces acting against the spores are drag, with additional input in repulsion and attraction from electrostatic forces [11]–[13].

Despite the importance of ballistospores for agriculture and human health, and the fascinating underlying cell biology and biophysics, no gene is known that affects ballistospore production or release. Two challenges in understanding ballistospores are that (*a*) rust fungi cannot be cultured away from a host plant and (*b*) the fruiting bodies of mushrooms contain two nuclei, limiting the employment of mutant screens for gene discovery since most mutations are likely to be recessive loss-of-function when compared to the wild type allele in the other nucleus. In contrast, the red yeasts are haploid, can be cultured easily in vitro, and generate ballistospores. We and others have recently developed transformation protocols for red yeasts, including a species of ballistospore-shooting *Sporobolomyces* [14]–[17], providing the means to dissect the gene functions in this group of organisms. In this study, we apply these tools towards understanding the genetic requirements of ballistospores formation and release. We report a mutant screen to identify strains with impaired shooting, and the identification and characterization of the first gene required for ballistospore production, demonstrating the requirement of very long chain fatty acids (VLCFAs) in this process.

## Materials and Methods

### Strains and transformation

The *Sporobolomyces* sp. *ura5* uracil auxotroph AIS2 was transformed using *Agrobacterium*-mediated delivery of T-DNA carrying the wild type *URA5* gene [14]. Strains were maintained on yeast extract peptone dextrose medium (YPD). Impaired ballistospore production or release was assessed by inoculating transformants on YPD medium in 10 mm deep Petri dishes, and inverting these over an uninoculated YPD plate. The wild type strain produces a mirror image on the uninoculated plate, whereas *mirror* mutants do not. The *URA5* marker was recycled by selection for mutations within it by plating strain GI209 on medium containing 1 g/L 5-fluoroorotic acid. The *S. cerevisiae phs1::KanMX*/*PHS1* heterozygote “magic marker” strain (Thermo Scientific Open Biosystems, Waltham, MA [18]) was transformed with plasmids as described below. Transformed strains were sporulated in 1% potassium acetate 0.005% zinc acetate, and plated onto magic medium to select for haploid strains carrying the *phs1::KanMX* allele [19]. Cafenstrole (analytical standard, Sigma-Aldrich, St. Louis, MO) was dissolved in DMSO and added to plates in one set of experiments. The fungal strains and their genotypes are listed in table 1.

**Table 1:** Strains used in this study.

Name	Genotype*	Background	Reference
***Sporobolomyces*** ** sp.**			
IAM 13481	Wild type		[41]
AIS2	*ura5*	IAM 13481	[14]
GI209	*phs1::URA5 ura5*	AIS2	This study
AIS11	*phs1::ura5 ura5*	GI209	This study
AIS13	*URA5 phs1::ura5 ura5*	AIS11	This study
AIS15	*PHS1-URA5 phs1::ura5 ura5*	AIS11	This study
***Saccharomyces cerevisiae***			
BY4741	*his3 leu2 met15 ura3*		[42]
YSC 4034-97/041153	*MAT*a/α *ura3 leu2 his3 lys2*/*LYS2 met15*/*MET15 can1::LEU2-MFA1pr*-*HIS3*/*CAN1 phs1::KanMX*/*PHS1*	RESGEN KanMx YKO heterozygous knock out strain + pXP346	[19], [43]
AIY6	*PHS1*/*phs1* + pTH19	YSC 4034-97/041153	This study
AIY9	*PHS1* + pTH19	BY4741	This study
AIY5	*PHS1*/*phs1* + pTH19-PHS1	YSC 4034-97/041153	This study
AIY8	*phs1::KanMX* + pTH19-PHS1 *lys2*	AIY5	This study
R1158	*URA3::CMV-tTA*	BY4741	[26]
TH-3237	*pTetO_7_-PHS1*	R1158	[28]

*Unless specified, the genotype of the background strain is also present in the strain, and is not re-written.

### DNA manipulation

Inverse PCR was used to amplify flanks of the T-DNA in *Sporobolomyces* as described previously [14]. The amplicons were sequenced and compared to the *Sporobolomyces* genome database (http://genome.jgi-psf.org/Sporo1/Sporo1.home.html) by BLASTn.

The ends of the *PHS1* gene were defined by 5′ and 3′ rapid amplification of cDNA ends (RACE) using the GeneRacer kit (Invitrogen, Life Technologies, Grand Island, NY) with gene specific primers (RA004 5′-ATGTGCGACATCATGAAGGC-3′ and RA009 5′-AGACCAGGCGGTAACCATC-3′ for 5′ RACE, and RA010 (5′-ATGTCTGCCGCTTCGCGC-3′) and ALID1374 5′-CTGCGTCGTCTTCCCTCAGGTCCCTC-3′ for 3′ RACE). The RNAs used for RACE were purified with TRIzol reagent (Invitrogen) from overnight cultures grown in yeast nitrogen base +2% dextrose. The full-length *PHS1* cDNA was amplified with primers RA010 (5′-ATGTCTGCCGCTTCGCGC-3′) and RA011 (5′-TCAGTGAGACGAAGTGATC-3′), and cloned into plasmid pCR2.1 (Invitrogen) to produce plasmid pRA1. The cDNA was subcloned with EcoRI into pTH19 [20], a 2 µ circle plasmid for replication in *S. cerevisiae* and that expressed the insert from the *ADH1* promoter. For complementation of the *phs1* mutation, a wild type copy of the gene was amplified with primers ALID1288 (5′-CCGCTCGAGCTGTCCTCAGAATGGTCTCG-3′) and ALID1289 (5′-CCGCTCGAGACATTTGACAGGCTTGGTCG-3′), digested with XhoI, and cloned into the XhoI site of plasmid pAIS4 [14]. The plasmid was electroporated into *A. tumefaciens* strain EHA105, and the *PHS1-URA5* T-DNA or the empty *URA5* T-DNA from pAIS4 trans-conjugated into *Sporobolomyces* strain AIS11.

### Lipid characterization

Sphingolipid metabolism and labeling experiments in *Sporobolomyces* strains and *S. cerevisiae* followed those as reported previously [21]. The elongation assay was performed as reported [22], [23].

### Stress sensitivity tests

For sensitivity tests, *mirror* mutants were serially diluted 10-fold in liquid YPD and spotted onto solid YPD agar containing caspofungin, congo red, fluconazole, hydrogen peroxide, sodium chloride, or sodium dodecyl sulfate. Non-shooting mutants were also exposed to UV irradiation and to 37°C for 7 h. Experiments were replicated three times.

## Results

### Identification of *mirror* mutants by T-DNA insertional mutagenesis

To identify factors that influence that ability of fungi to produce or shoot ballistospores, a forward genetic screen was used in a species of *Sporobolomyces* (strain IAM 13481). Approximately 5,000 T-DNA insertional mutants were generated using *Agrobacterium*-mediated transformation. The transformants were patched on agar medium to form colonies, and inverted over uninoculated plates of media. 18 *mirror* mutants were identified with loss or reduction of the ability to transfer to the bottom plate (Fig. 1C).

Examination of the colonies of the 18 strains by microscopy revealed that the most common reason (17 cases) for the inability to produce the mirror image was the loss or large reduction in the ability to produce the ballistospore. One strain, GI277, still produced ample ballistospores, but was unable to release them (Fig. S1).

**Figure 2:**
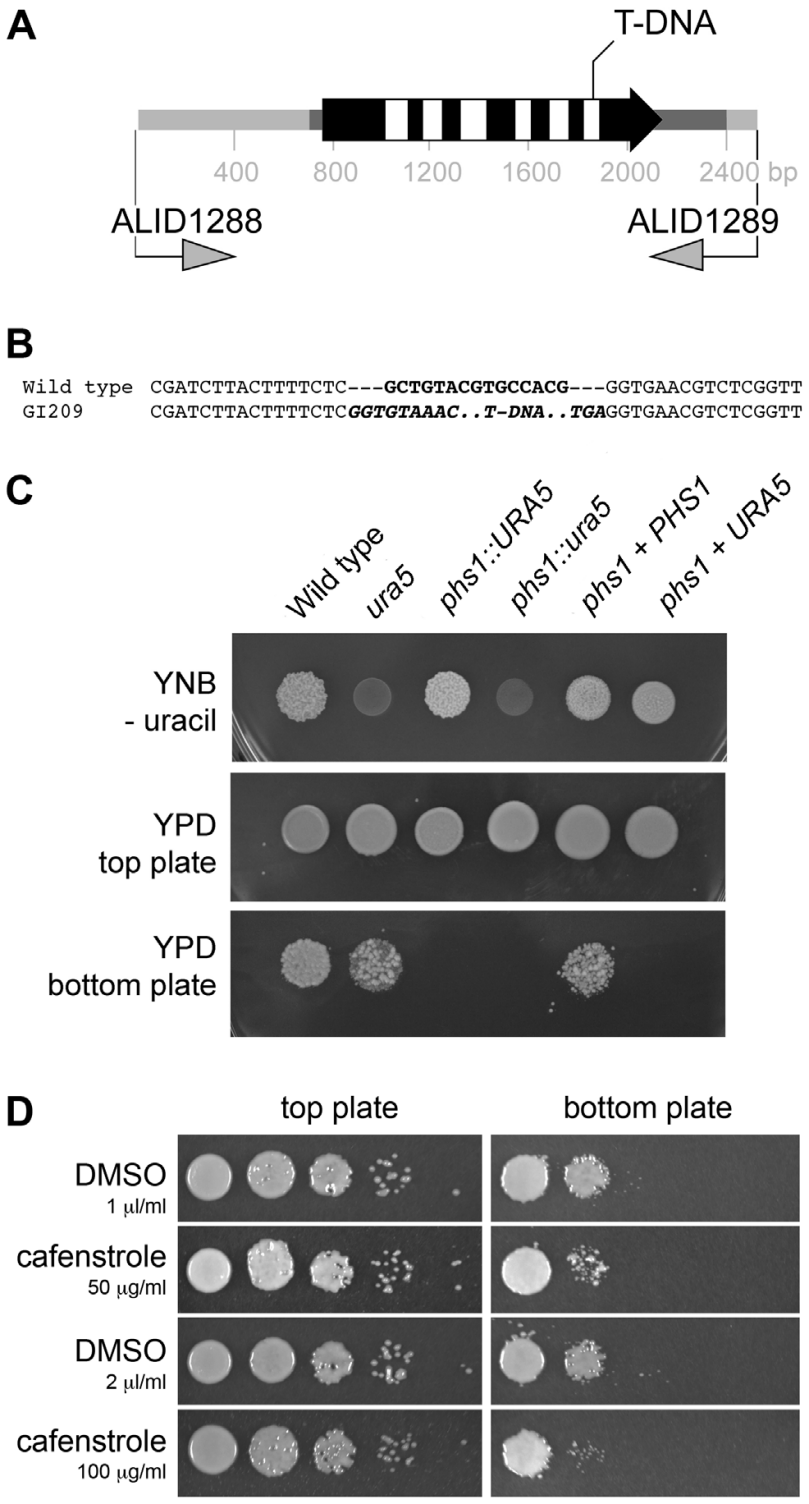
Loss of *PHS1* impairs the ability to produce ballistospores. (A) The structure of the *PHS1* gene, and the T-DNA insertion position within it in strain GI209. Exons are in black and introns in white, with the 5′ and 3′ untranslated regions in darker grey. The two primers, ALID1288 and ALI1289, were used to amplify the wild type copy for gene complementation. (B) DNA sequence alignments of the wild type or T-DNA insertional mutant strain. The 15-bp black region in the wild type is replaced with the T-DNA molecule, whose left and right border sequences are in bold italics. (C) The loss of *PHS1* can be complemented by reintroducing a wild type copy of the *PHS1* gene. Yeast nitrogen base (YNB) without uracil is the selection medium for transformation. Ballistospores were fired from the top plate onto the bottom plate, and colonies allowed to grow. (D) An inhibitor of VLCFA synthesis, cafenstrole, reduces ballistospore production. Wild type *Sporobolomyces* were 10-fold serially diluted, and grown on YPD supplemented with cafenstrole or the solvent DMSO for two days (top plate) inverted over the bottom plates. The bottom plates were cultured two additional days.

Strain GI209, with reduced spore production and with a single T-DNA insertion in its genome as based on Southern blot analysis, was chosen for further study. The junctions of its T-DNA insertion were obtained by inverse PCR and compared by BLASTn to the genome sequence database of *Sporobolomyces*. The T-DNA inserted close to a gene annotated as a *PHS1* homolog, that encodes 3-hydroxyacyl-CoA dehydratase for the third step in very long chain fatty acid biosynthesis (Fig. 2).

### A *phs1* mutant has reduced ballistospore production

The structure of the *PHS1* gene in *Sporobolomyces* was ambiguous in the genome sequencing database. Based on BLAST comparisons, we predicted the gene extended beyond the annotated version, and that the T-DNA insertion would lie within the final intron of the gene to generate an allele with residual function. The 5′ and 3′ ends of the *PHS1* transcript were determined by rapid amplification of cDNA ends, allowing us to annotate this gene correctly and confirm the hypothesis that the T-DNA insertion in strain GI209 is in the final intron (Fig. 2A, B). A 15-bp deletion following the T-DNA insertion event also occurred (Fig. 2B). The strain GI209, *ura5 phs1::URA5,* is indicated in the text and in the figures as *Sporobolomyces* mutant *phs1*, with the exception of figure 2B where it is indicated as *phs1::URA5*. The *PHS1* gene sequence is deposited in GenBank as accession JQ068150.

To show that the phenotype of strain GI209 is due to mutation of *PHS1*, functional complementation was carried out. Due to the lack of gene markers in *Sporobolomyces*, the complementation reported here is based on a uracil marker recycling system. An uracil auxotroph, AIS11, was isolated from strain GI209, by selection on YNB +5-FOA, and transformed by *A. tumefaciens* either with the *URA3* or *URA5* gene of *Sporobolomyces*. The *URA5* gene complemented the mutation, indicating that AIS11 is *ura5 phs1::ura5*. This strain is indicated in figure 2B as *phs1::ura5*. One stable *ura5 phs1::ura5 URA5* transformant, AIS13, was used as a control for further experiments; this strain is indicated in the text and in the figures as *phs1* + *URA5*. For complementation, the wild type copy of the *PHS1* gene was amplified from *Sporobolomyces*, cloned in the plasmid pAIS4 containing the *Sporobolomyces URA5* gene, and introduced by *A. tumefaciens* into the uracil auxotroph AIS11. One stable *ura5 phs1::ura5 PHS1 URA5* transformant, AIS15, was selected; this strain is indicated in the text and in the figures as *phs1* + *PHS1*. In strain AIS15, the wild type gene complemented the *mirror* mutant phenotype, providing evidence that the mutation in *PHS1* causes the loss of ballistospore production (Fig. 2C).

There are several possible consequences to transcript production of having the T-DNA insertion in the final intron of *PHS1* in strain GI209. The 3′ end for the transcript produced by this mutant allele was sought by RACE and amplicons cloned and sequenced. This revealed at least one scenario is that the original 5′ splice site still functions, but a new 3′ splice site within the T-DNA sequence is used. Further, a second and novel intron within the T-DNA is also spliced. This information is illustrated as Fig. S2.

The chloroacetamides are a class of chemicals used in agriculture for weed control, and that act by inhibition of VLCFA synthesis [24], [25]. Cafenstrole was tested on the wild type *Sporobolomyces* strain. At concentrations that allowed equal growth compared to the solvent control, the ability to mirror to the bottom plate was reduced almost an order of magnitude (Fig. 2D). This result provides additional evidence that VLFCA synthesis is important for the proper formation of ballistospores.

### The *Sporobolomyces PHS1* gene complements the lethality of deleting *PHS1* in *Saccharomyces cerevisiae*

**Figure 3:**
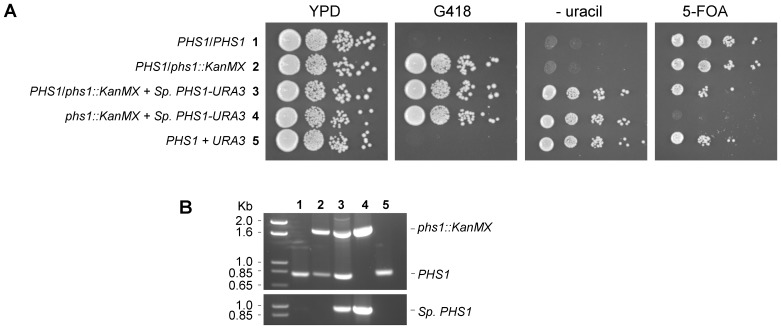
*Sporobolomyces PHS1* complements loss-of-function of the *S. cerevisiae phs1* homolog. Five strains (numbered 1–5) were cultured on different media (A) and the presence or absence of the native *PHS1* or the *Sporobolomyces PHS1* gene in *S. cerevisiae* tested by PCR (B). (A) Ten fold serial dilutions of yeast strains. YPD allows equal growth of the strains. G418 selects for strains carrying a *phs1::KanMX* allele. The YNB – uracil selects for strains carrying a plasmid with the *URA3* selectable marker. 5-FOA is used to counter-select or “cure” plasmids containing the *URA3* marker from a *ura3* mutant strain background. Strain 1 is a diploid with both copies of *PHS1*. Strain 2 is a heterozygote strain with one wild type and one mutated copy of the *PHS1* gene. Strain 3 is strain 2 expressing the *Sporobolomyces PHS1* gene from a 2 µ plasmid. Strain 4 is a haploid progeny from meiosis of strain 3. Strain 5 is a control haploid strain with the 2 µ plasmid. (B) The top panel shows PCR of the wild type allele of *S. cerevisiae PHS1* (near the 0.85 kb ladder marker) or the replacement allele *phs1::KanMX* (near to 1.6 kb ladder marker). The bottom panel is amplification of the *Sporobolomyces PHS1* gene from the same strains.

Comparison of the *Sporobolomyces* Phs1 amino acid sequence with its characterized homologs that are available in GenBank suggests that the protein would function in VLCFA biosynthesis. For instance, alignment of the homolog with the Phs1 protein sequence of *S. cerevisiae* reveals that all amino acid residues known to be important for function are conserved in the *Sporobolomyces* protein [[26], [27] Fig. S3]. To infer similar biochemical function, we tested if the *Sporobolomyces* gene could complement the loss of the *PHS1* gene from *S. cerevisiae*, where it is essential for viability. The *Sporobolomyces* cDNA was cloned into a yeast expression plasmid, and the plasmid introduced into a *phs1*/*PHS1* heterozygote strain of *S. cerevisiae*. As a control, the empty plasmid was also transformed into this *phs1*/*PHS1* heterozygote strain. After meiotic sporulation and selection for the haploids [19], the strains transformed with the *Sporobolomyces PHS1* gene yet carrying a deletion of the *S. cerevisiae* homolog were able to grow, whereas no progeny were obtained for the strain carrying the empty plasmid (Fig. 3A). This was further supported by the observation that plasmids could be “cured” from strains by selection on medium containing 5-FOA that is toxic to strains carrying a functional *URA3* gene, except in the case of the *phs1::KanMX* haploid strain (Fig. 3A). The absence or presence of the *PHS1* genes in the *S. cerevisiae* strains was confirmed by PCR analysis (Fig. 3B). These suggest that the *Sporobolomyces PHS1* gene has the same functions as the *S. cerevisiae* homolog.

### Mutation of *PHS1* in *Sporobolomyces* sp. impairs 3-hydroxyacyl-CoA dehydratase activity and alters the lipid profiles of the cells

**Figure 4:**
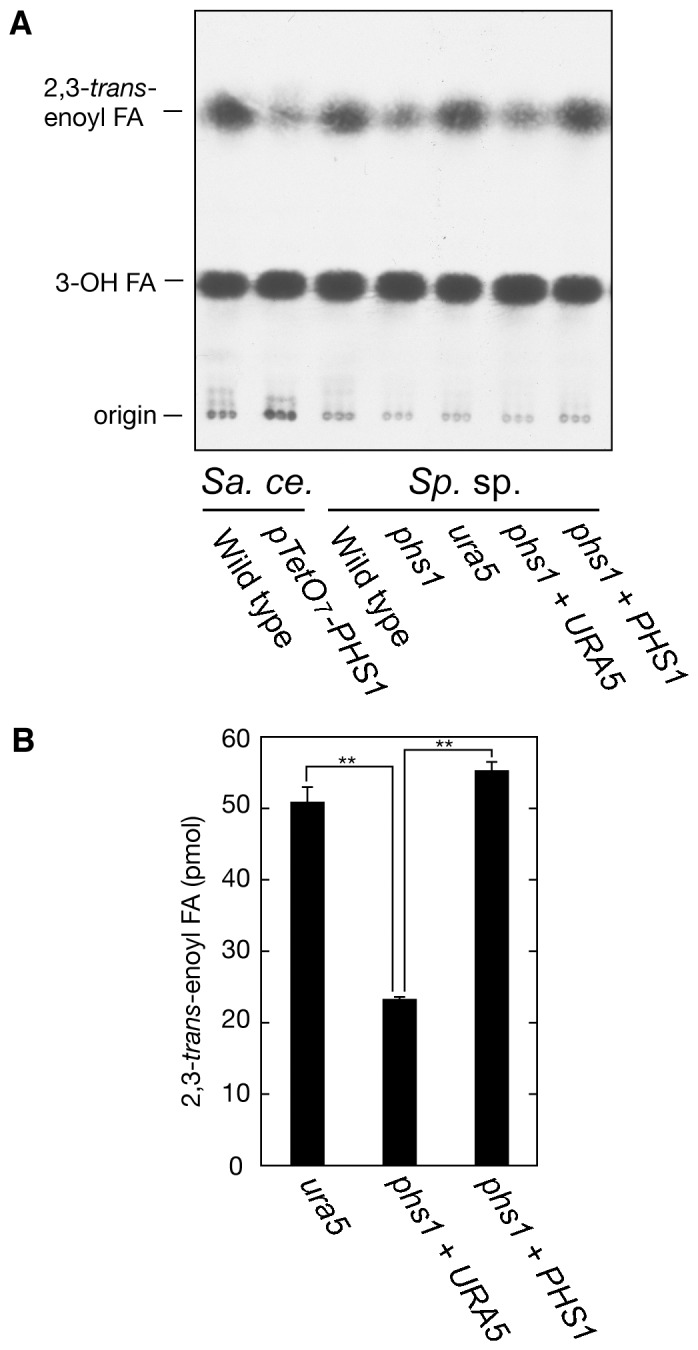
The *phs1* mutation in *Sporobolomyces* causes reduction in 3-hydroxyacyl-CoA dehydratase activity. (A) *Sporobolomyces* (*Sp*. sp.) wild type (IAM 13481), mutant *phs1* (GI209), *ura5* auxotroph (AIS2), uracil prototroph *phs1+ URA5* (AIS13), and complementing strain *phs1 + PHS1* (AIS15) cells were grown in YPD medium at 25°C. As controls, *S. cerevisiae* (*Sa. ce.*) wild type (R1158) and *pTetO_7_-PHS1* (TH-3237) were grown in YPD medium containing 10 µg/ml doxycyclin for 6.5 h at 30°C. Total membranes (4 µg) prepared from the strains were incubated for 15 min at 37°C [^14^C]3-hydroxypalmitoyl-CoA (3.6 µM; 10 nCi/µl). After termination of the reactions, lipids were saponified, acidified, extracted, and separated by normal phase TLC. (B) The radioactivities associated with the reaction product 2,3-*trans* hexadecenoic acid were quantified using a bioimaging analyzer BAS-2500. Values represent the mean ± S.D. from three independent experiments. Statistically significant differences are indicated (**p<0.01; t-test).

The second evidence for *Sporobolomyces* Phs1 being a 3-hydroxyacyl-CoA dehydratase is altered enzymatic activity in the T-DNA *phs1* mutant GI209. Membranes were extracted from *Sporobolomyces* strains and *S. cerevisiae* control strains. The two *S. cerevisiae* strains, R1158 (wild type) and TH-3237 (*pTetO_7_-PHS1*), were used as controls since their activities and lipid profiles have been characterized previously. TH-3237 cells carry the *PHS1* gene under control of the *TetO_7_* promoter. Doxycyclin can shut off expression [26], [28]. The extracts were incubated with [^14^C]3-hydroxypalmitoyl-CoA, then lipids processed and separated by normal phase thin layer chomatography. The radioactivities associated with the reaction product 2,3-*trans* hexadecenoic acid were compared between strains. In mutants *phs1* and *phs1*+ *URA5*, the T-DNA insertion caused a reduction in the 3-hydroxyacyl-CoA dehydratase activity to about half compared to the wild type cells of IAM 13481 and AIS2 (*ura5*), or the complemented control strain AIS15 (*phs1 + PHS1*). Interestingly, the reduction in 2,3-*trans* hexadecenoic acid levels in the *Sporobolomyces* mutant was equivalent to that seen in the *PHS1*-repressed strain of *S. cerevisiae* (Fig. 4A). Furthermore, the radioactivity associated with the reaction product 2,3-*trans* hexadecenoic acid was significantly higher in strains bearing a functional *PHS1* (strains AIS2 and complemented *phs1 + PHS1*) compared to the *phs1* mutant (Fig. 4B).

**Figure 5:**
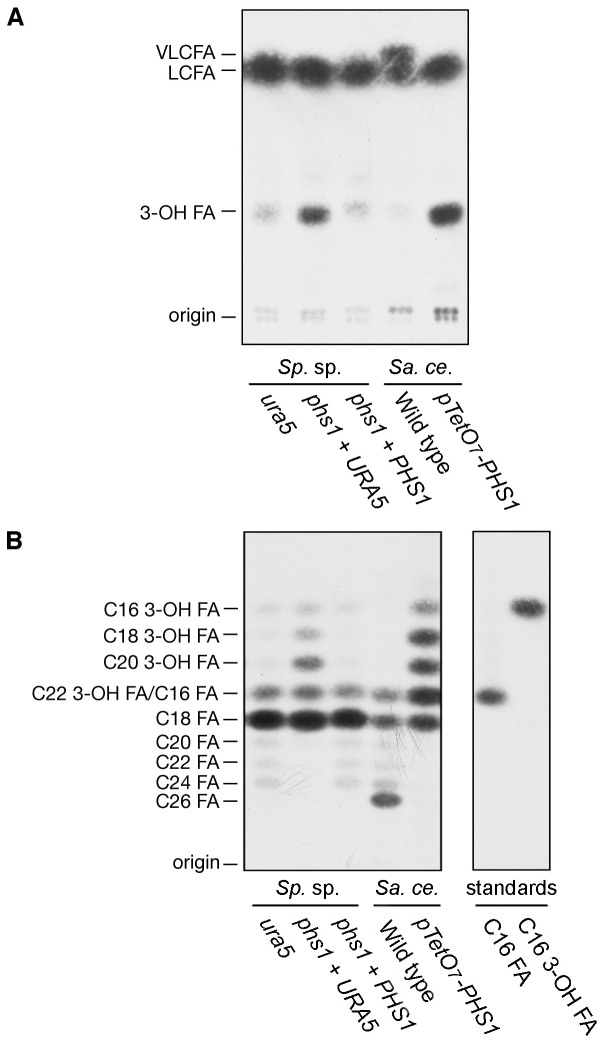
The *phs1* mutation causes reduction in elongation of C16:0-CoA. (A) *Sporobolomyces* (*Sp*. sp.) *ura5* auxotroph (AIS2), uracil prototroph *phs1*+ *URA5* (AIS13) and complementing strain *phs1 + PHS1* (AIS15) cells were grown in YPD medium at 25°C. *S. cerevisiae* (*Sa. ce.*) wild type (R1158) and *pTetO_7_-PHS1* (TH-3237) strains were grown in YPD medium containing 10 µg/ml doxycyclin for 6.5 h at 30°C. Total membranes (20 µg) prepared from the strains were incubated with C16:0-CoA (20 µM) and 100 µM (0.075 µCi) [^14^C]malonyl-CoA in the presence of 1 mM NADPH for 1 h at 37°C. (A) After termination of the reactions, lipids were saponified, acidified, extracted, and separated by normal-phase TLC, followed by detection by autoradiography. (B) After termination of the reactions, lipids were saponified, acidified, and extracted. The resulting fatty acids were then converted to fatty acid methyl ester, extracted, separated by reverse-phase TLC, and detected by autoradiography. [^3^H]Palmitic acid (C16 FA) and [^14^C]3-hydroxypalmitic acid (C16 3-OH FA) were used as standards.

A fatty acid elongation assay was used with the substrates palmitoyl-CoA and malonyl-CoA in the presence of NADPH. In wild type *S. cerevisiae* R1158, C16-CoA was elongated to C26-CoA (Fig. 5) as previously reported. In the engineered *S. cerevisiae* strain TH-3237, *pTetO_7_-PHS1*, a differential spot corresponding to 3-hydroxy acyl-CoAs (3-OH FA) was detected (Fig. 5A). Separation of the reaction products by reverse phase TLC revealed that the chain-lengths of the accumulated 3-hydroxyacyl-CoAs were a mixture of C16, C18, C20, and C22 (Fig. 5B). In strains of *Sporobolomyces* having a wild type copy of the *PHS1* gene, C16-CoA was mainly elongated to C18-CoA and slightly to C24-CoA, similarly to the wild type of *S. cerevisiae* R1158 with the exception of C26-CoA. In *Sporobolomyces phs1* + *URA5*, 3-hydroxyacyl-CoAs accumulated like those in the *pTetO_7_-PHS1 S. cerevisiae* cells (Fig. 5A and B), further confirming the role of the *Sporobolomyces PHS1* gene in the elongation of VLCFAs.

**Figure 6:**
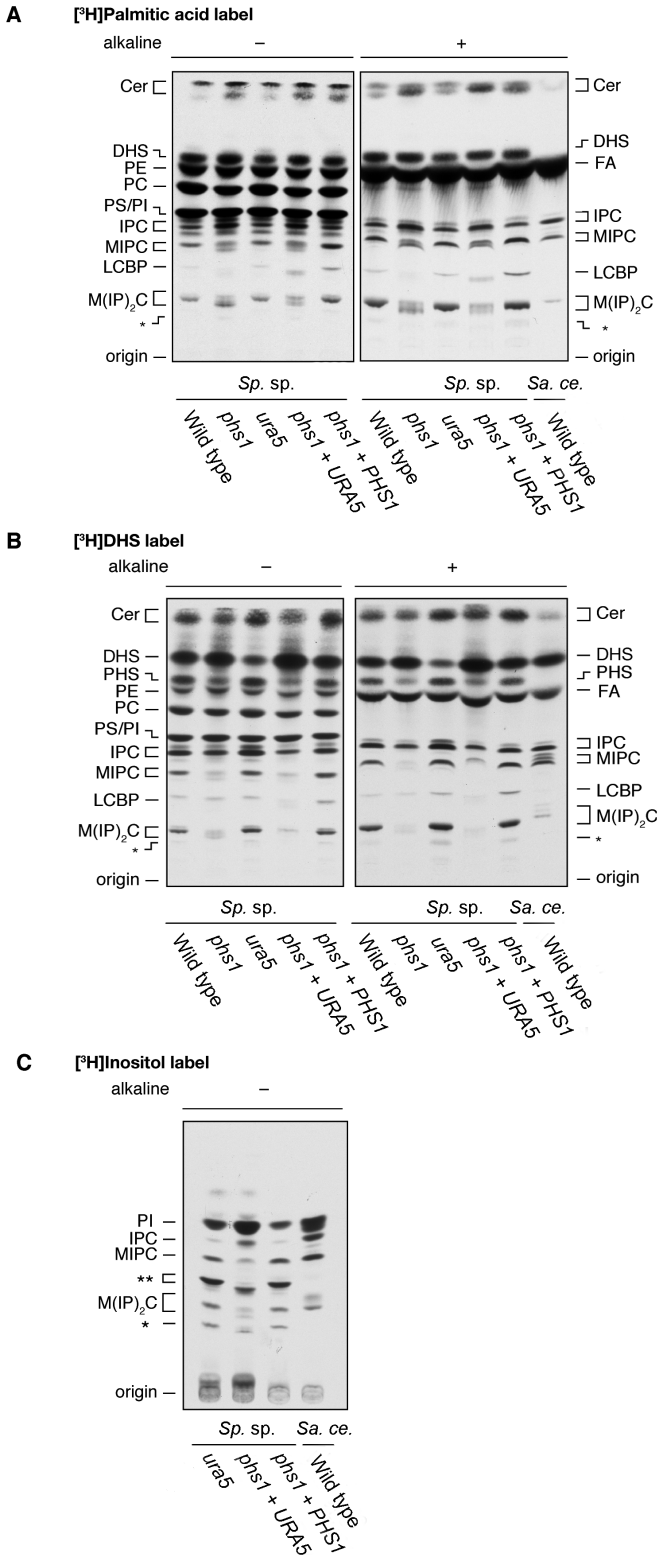
The *phs1* mutation affects sphingolipid synthesis. *Sporobolomyces* (*Sp*. sp.) wild type (IAM 13481), mutant *phs1* (GI209), *ura5* auxotroph (AIS2), uracil prototroph *phs1*+ *URA5* (AIS13), and complementing strain *phs1 + PHS1* (AIS15) cells and *S. cerevisiae* (*Sa. ce.*) wild type (R1158) were grown in YPD medium medium (A and B) or in SC medium lacking inositol (C) at 25°C. Cells were labeled with [^3^H]palmitic acid (A), [^3^H]dihydrosphingosine (B), or [^3^H]inositol (C) for 2 h. Lipids were extracted, treated with nothing or alkaline solution, and separated by normal phase TLC, followed by detection by autoradiography. Abbreviations are CER, ceramide; DHS, dihydrosphingosine; FA, fatty acid; PE, phosphatidylethanolamine; PHS, phytosphingosine; PC, phosphatidylcholine; PS, phosphatidylserine; PI, phosphatidylinositol; IPC, inositolphosphorylceramide; MIPC, mannosylinositol phosphorylceramide; LCBP, long chain base phospholipid; M(IP)2C, mannosyldiinositol phosphorylceramide. The asterisks indicate unidentified lipids.

Eukaryotes synthesize a suite of VLCFAs or derived molecules. The fungal strains were cultured in [^3^H]palmitic acid, [^3^H]dihydrosphingosine, or [^3^H]inositol, the lipids extracted and resolved by normal phase TLC. *Sporobolomyces* likely contains the same sphingolipids as *S. cerevisiae*, that is IPC, MIPC, and M(IP)_2_C (Fig. 6). We speculate that the fatty acid portion of sphingolipids in *Sporobolomyces* may be C18 or C24, but not C26. The T-DNA insertion into the *phs1* gene caused reductions in MIPC and M(IP)_2_C but an increase in IPC. Synthesis of glycerophospholipids was not affected. MIPC and M(IP)_2_C in the *phs1* mutants migrated slower than those in the wild type strain on TLC plates. It is possible that MIPC and M(IP)_2_C in the *phs1* mutants contain an abnormal hydroxy group or a shorter fatty acid compared to *S. cerevisiae*. The asterisks in Fig. 6 indicate unidentified lipids. One could be a M(IP)_2_C derivative, such as M(IP)_2_C containing an additional hydroxy group or sugar.

### Additional phenotypes are associated with mutation of *PHS1* and other *mirror* mutants

**Figure 7:**
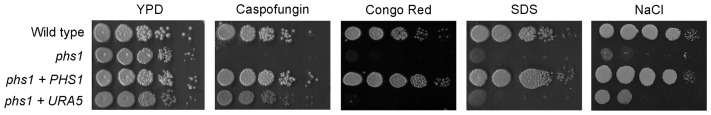
Stress phenotypes of the *phs1* mutant of *Sporobolomyces* suggest that defects in other cellular processes can impair the formation of spores. 10-fold serial dilutions of overnight cultures of *Sporobolomyces* wild type (IAM 13481), mutant *phs1* (GI209), complementing strain *phs1 + PHS1* (AIS15) and uracil prototroph *phs1+ URA5* (AIS13) were plated onto YPD media without or with stress agents, and cultured 3–4 days. Stress agents were caspofungin (50 µg/ml), congo red (0.4%), sodium dodecyl sulfate (SDS, 0.015%) and sodium chloride (NaCl, 1 M).

VLCFAs are associated with a number of cellular processes. We expected that additional phenotypes should be observed for the *phs1* T-DNA insertion mutant, and compared its growth under different conditions, along with the other 17 *mirror* mutants for comparison. Of the 18 strains examined, 14 also showed impaired growth under one or more stress conditions (Fig. S1B). In the case of the *phs1* mutant, it showed strong sensitivity to agents like the Fks1 inhibitor caspofungin, congo red, sodium chloride and the detergent sodium dodecyl sulfate (Fig. 7). Caspofungin and congo red act by inhibiting the enzyme (1, 3)-β-D-glucan synthase to disturb the integrity of the fungal cell wall; sodium dodecyl sulfate affects membrane stability and also, indirectly, cell wall construction; and NaCl interferes with the normal osmolarity of the cells, thus damaging cells with cell wall defects. The re-introduction of the wild type copy of the *PHS1* gene into the mutant *phs1* restored the tolerance to the stress compounds back to the wild type level, indicating additional roles of the *Sporobolomyces PHS1* gene in cell wall function and maintenance. This information suggests that some genes, such as *PHS1*, required for ballistospore production or release from the parent yeast cell also have other roles in the cell.

## Discussion

Ballistospores are a mobilized spore type found in one lineage of the fungi, the Basidiomycota. Although these spores play important roles in life cycles of these fungi, no gene involved in their biosynthesis or release has been characterized. In part this is due to the technical challenges in working with rust fungi or mushrooms. As a first step towards identifying factors that are required for ballistospore formation or release, we performed a genetic screen on a collection of mutants. The screen is far from saturated (5,000 transformants; the *Sporobolomyces* sp. IAM 13481 genome project estimates 5,500 genes). 18 transformants (3.6%) were isolated as impaired in efficient spore shooting, being unable to make a mirror image of the colonies onto an adjacent Petri dish. Characterization of other phenotypes in these mutants suggests that many are affected in cell wall, membrane or polarity in generating the ballistospores, because the strains are also sensitive to agents that cause such stress.

We characterized one *Sporobolomyces* strain bearing a mutation in the *PHS1* gene in detail: this gene encodes the 3-hydroxyacyl-CoA dehydratase enzyme required for VLCFA biosynthesis. The VLCFAs have chain lengths of more than 20 carbons. These molecules are synthesized by a four-step process of sequential elongation, that add two carbons to the chain for each cycle. The pathway for VLCFA synthesis is conserved in eukaryotes and it plays essential roles in physiology related to lipid membranes [23]. The importance of this pathway is illustrated by the factor that loss of VLFCA synthesis is lethal in fungi like *S. cerevisiae* or *S. pombe* [29]. *PHS1* first emerged in studies in *S. cerevisiae*, where genetic screens revealed that it was essential for cell viability [30]–[32]. At about the same time, mutant screens in the plant *Arabidopsis thaliana* identified a gene required for cell differentiation and proliferation. Mutants form callus tissue instead of leaves and stems, and the phenotype is exasperated by addition of cytokinin plant hormones. The *PASTICCINO2* (or *PEPINO*) gene was subsequently cloned, and is able to complement the *S. cerevisiae phs1* mutation [33]–[36]. Key studies elucidating the function of Phs1 were conducted in *S. cerevisiae*, showing that the protein catalyzes the third step in biosynthesis of very long chain fatty acids [26], [37]. Human and plant homologs have similar activities [28], [38]. Recently, a human inbred family has been identified with childhood myopathogies attributable to a mutation in one of the four human homologs of *PHS1*, *HACD1* [39]. The identity of this human gene was in part guided by a dog centronuclear myopathy model, which is linked to a transposon insertion in the homolog of this gene [40].

The isolation of the *Sporobolomyces* allele of *phs1* in this study was fortuitous since the gene is essential in other fungi. The T-DNA insertion is within the final intron, past the essential amino acid residues required for function. Characterization of the transcript produced in the strain revealed a chimeric form that utilized information within the T-DNA region, including for splicing novel introns. We speculate that this aberrant protein maintains some residual hydroxyacyl-CoA dehydratase activity. The mechanism by which reduction of Phs1 function impairs ballistospore formation is currently unknown, but likely is by alteration of cell polarity, membrane properties, or vesicle transport. Overall, the identification of the VLCFA pathway as a requirement for ballistospore formation adds another striking property to the growing list of important biological processes – Phs1 is essential in yeasts, regulates differentiation and division in plants, and impacts muscle function in mammals – controlled by these molecules.

This study demonstrates that the genes for ballistospores no longer need to remain unknown. Future developments in molecular tools in *Sporobolomyces* sp. or other ballistospore-forming yeasts, and the identification of genes affected in other mutants, will reveal insights into this unique cell biology process. Of particular interest are mutants that can produce ballistospores, but are unable to fire them. The identification of the genes required for ballistospore formation may also have practical applications. For instance, chemical reagents that inhibit VLCFA biosynthesis or the development of drugs targeting genes involved in sporulation could be used in control of rust fungi on infected crops or their alternative host plants, or to reduce allergenic spores to improve the indoor environmental quality in office buildings, schools, and other non-industrial buildings.

## Supporting Information

Figure S1 **Summary of spore production and the sensitivity tests to cell wall and plasma membrane stress-generating compounds of non-shooting mutants of** ***Sporobolomyces*** **.** (A) Images of the surface of colonies of the wild type or *mirror* mutant strains. The photographs were modified to provide contrast such that each dark dot represents a ballistospore. (B) Ten-fold serial dilutions of yeast cells were spotted onto YPD without any supplements or containing caspofungin (50 µg/ml), congo red (0.4%), fluconazole (FLC, 32 and 64 µg/ml), hydrogen peroxide (H_2_O_2_, 2 mM), sodium chloride (NaCl, 1 M), sodium dodecyl sulfate (SDS, 0.015%). The strains were also exposed to UV irradiation (120 J/m^2^) and to 37°C for 7 h.
(TIF)

Figure S2 **Comparison of 3′ ends of the** ***PHS1*** **alleles from wild type and strain GI209.** Coding nucleotides are in upper case, with the difference in amino acid sequence between the predicted proteins encoded by the two alleles in green. Introns are in grey lower case, or orange for the two new intron sequences in strain GI209. Blue sequence represents 3′ untranslated regions (one intron in GI209 falls in this region). The underlined region is the T-DNA sequence.
(TIF)

Figure S3 **Alignment of** ***Sporobolomyces*** **Phs1 predicted amino acid sequence with characterized homologs.** The homologs are from *S. cerevisiae* (*PHS1*), human (*HACD1*) and *A. thaliana* (*PASTICCINO2*). Conserved sites with characterized functions are marked with green highlighted asterisks. Grey m letters above the black font indicate transmembrane residues. Amino acid residues in blue indicate cytoplasmic and in orange indicates ER lumen localization.
(TIF)
