# Supplementary figures and images for: Phs1 and the Synthesis of Very Long Chain Fatty Acids Are Required for Ballistospore Formation
Source: PLoS One. 2014 Aug 22;9(8):e105147. doi: 10.1371/journal.pone.0105147 (PMC4141788; doi:10.1371/journal.pone.0105147)

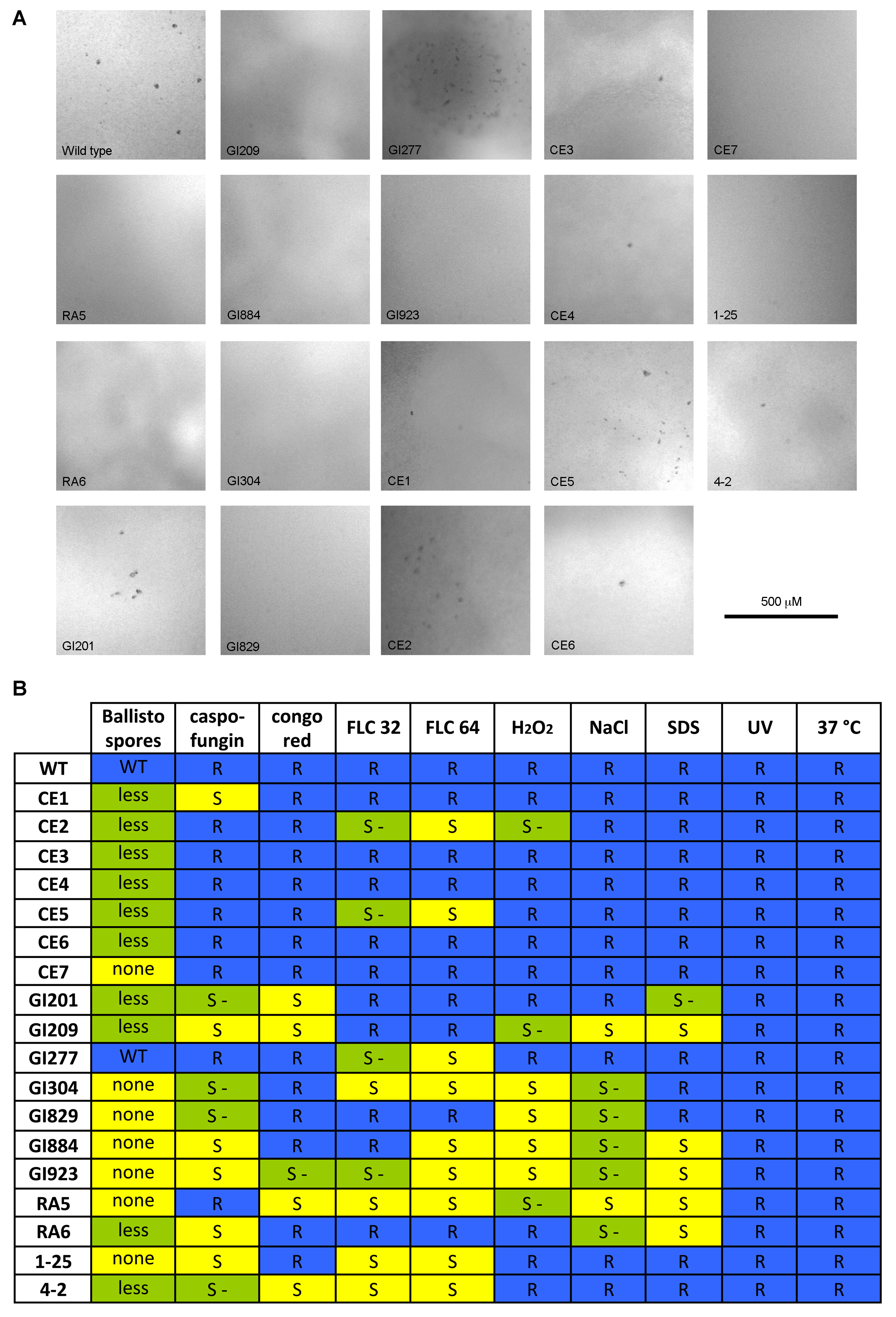

Supplement: Figure S1 — Summary of spore production and the sensitivity tests to cell wall and plasma membrane stress-generating compounds of non-shooting mutants of Sporobolomyces . (A) Images of the surface of colonies of the wild type or mirror mutant strains. The photographs were modified to provide contrast such that each dark dot represents a ballistospore. (B) Ten-fold serial dilutions of yeast cells were spotted onto YPD without any supplements or containing caspofungin (50 µg/ml), congo red (0.4%), fluconazole (FLC, 32 and 64 µg/ml), hydrogen peroxide (H2O2, 2 mM), sodium chloride (NaCl, 1 M), sodium dodecyl sulfate (SDS, 0.015%). The strains were also exposed to UV irradiation (120 J/m2) and to 37°C for 7 h. (TIF) [file pone.0105147.s001.tif]

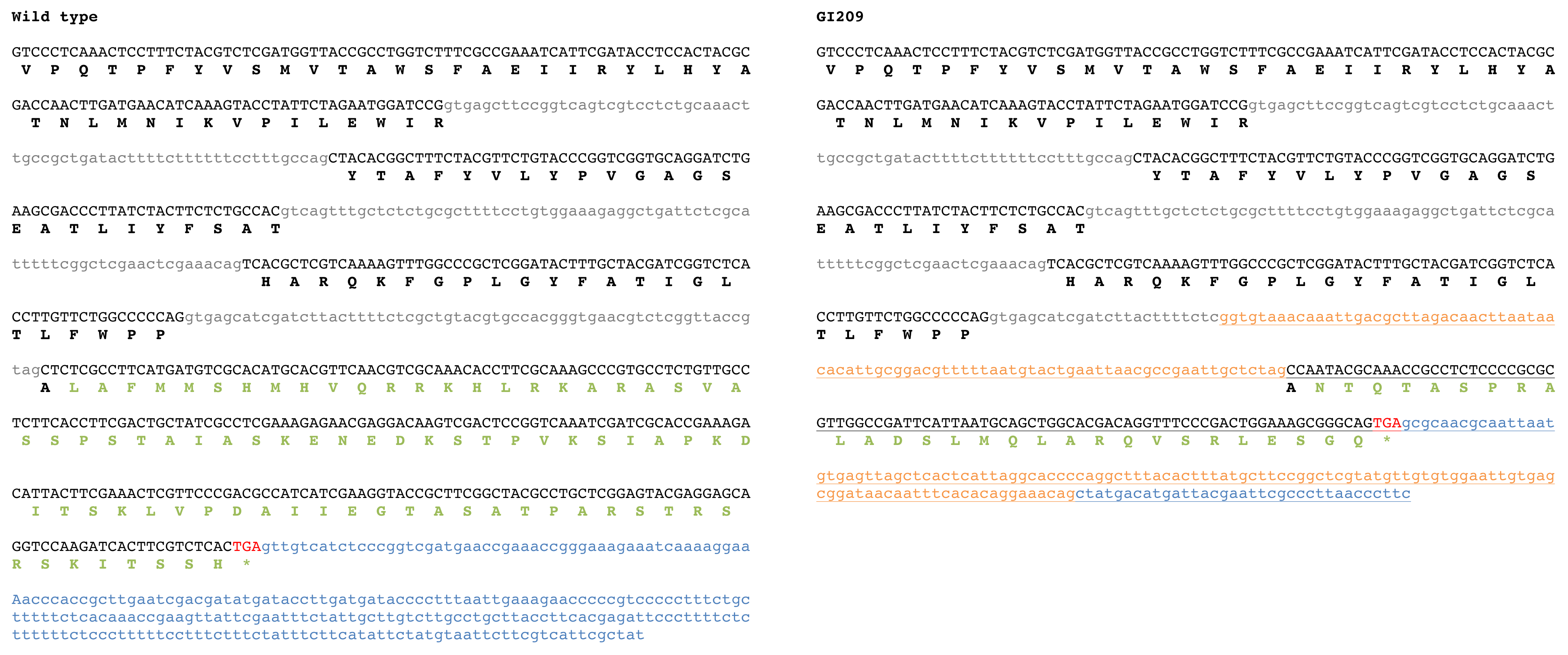

Supplement: Figure S2 — Comparison of 3′ ends of the PHS1 alleles from wild type and strain GI209. Coding nucleotides are in upper case, with the difference in amino acid sequence between the predicted proteins encoded by the two alleles in green. Introns are in grey lower case, or orange for the two new intron sequences in strain GI209. Blue sequence represents 3′ untranslated regions (one intron in GI209 falls in this region). The underlined region is the T-DNA sequence. (TIF) [file pone.0105147.s002.tif]

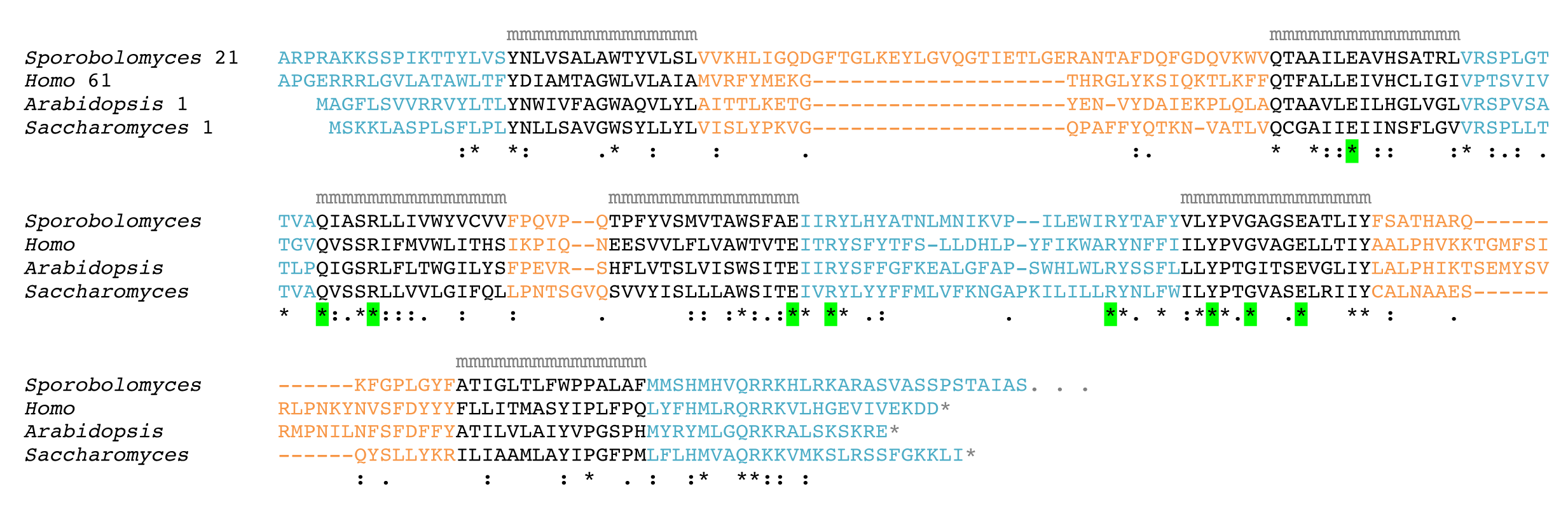

Supplement: Figure S3 — Alignment of Sporobolomyces Phs1 predicted amino acid sequence with characterized homologs. The homologs are from S. cerevisiae (PHS1), human (HACD1) and A. thaliana (PASTICCINO2). Conserved sites with characterized functions are marked with green highlighted asterisks. Grey m letters above the black font indicate transmembrane residues. Amino acid residues in blue indicate cytoplasmic and in orange indicates ER lumen localization. (TIF) [file pone.0105147.s003.tif]
